# A prospective evaluation of first people’s health promotion program design in the goulburn-murray rivers region

**DOI:** 10.1186/s12913-016-1878-4

**Published:** 2016-11-10

**Authors:** Joyce Doyle, Sharon Atkinson-Briggs, Petah Atkinson, Bradley Firebrace, Julie Calleja, Rachel Reilly, Margaret Cargo, Therese Riley, Tui Crumpen, Kevin Rowley

**Affiliations:** 1 The Onemda Group, Centre for Health Equity, Melbourne School of Population & Global Health, The University of Melbourne, Melbourne, VIC Australia; 2 Viney Morgan Aboriginal Medical Service, Barmah, VIC Australia; 3 Wardliparingga Aboriginal Research Unit, South Australian Health and Medical Research Institute, North Terrace, Adelaide, SA 5000 Australia; 4 School of Health Sciences, University of South Australia, Adelaide, SA Australia; 5 Centre of Excellence in Intervention and Prevention Science, Carlton, VIC 3053 Australia; 6 Goulburn Valley Partnerships Office, The University of Melbourne, Shepparton, VIC 3630 Australia

**Keywords:** Health Promotion, Community Based, Indigenous Peoples, Aboriginal and Torres Strait Islander people, Ecological, Evaluation

## Abstract

**Background:**

Aboriginal Community Controlled Organisations (ACCOs) provide community-focussed and culturally safe services for First Peoples in Australia, including crisis intervention and health promotion activities, in a holistic manner. The ecological model of health promotion goes some way towards describing the complexity of such health programs. The aims of this project were to: 1) identify the aims and purpose of existing health promotion programs conducted by an alliance of ACCOs in northern Victoria, Australia; and 2) evaluate the extent to which these programs are consistent with an ecological model of health promotion, addressing both individual and environmental determinants of health.

**Methods:**

The project arose from a long history of collaborative research. Three ACCOs and a university formed the Health Promotion Alliance to evaluate their health promotion programs. Local community members were trained in, and contributed to developing culturally sensitive methods for, data collection. Information on the aims and design of 88 health promotion activities making up 12 different programs across the ACCOs was systematically and prospectively collected.

**Results:**

There was a wide range of activities addressing environmental and social determinants of health, as well as physical activity, nutrition and weight loss. The design of the great majority of activities had a minimal Western influence and were designed within a local Aboriginal cultural framework. The most common focus of the activities was social connectedness (76 %). Physical activity was represented in two thirds of the activities, and nutrition, weight loss and culture were each a focus of about half of the activities. A modified coding procedure designed to assess the ecological nature of these programs showed that they recruited from multiple settings; targeted a range of individual, social and environmental determinants; and used numerous and innovative strategies to achieve change.

**Conclusion:**

First Peoples’ health promotion in the Goulburn-Murray Rivers region encompasses a broad range of social, cultural, lifestyle and community development activities, including reclaiming and strengthening cultural identity and social connectedness as a response to colonisation.

## Background

The Aboriginal community of the Goulburn-Murray Rivers Region has the largest population of First Peoples in Victoria, outside of the Melbourne metropolitan area, with several thousand Aboriginal and Torres Strait Islander people living or passing through the Shepparton region. The region includes the traditional lands of the Yorta Yorta and Bangarang nations. There are a large number of Aboriginal organisations operating and providing services to the community. These organisations are community focused and culturally safe, and play an important role in employment, education and health for First Peoples of the region. Aboriginal Community Controlled Organisations (ACCOs) such as these play an important role in providing culturally safe services, strengthening First Peoples’ identity, and in advocacy for health and human rights [1, 2]. ACCOs emerged from community in the 1970s as a means of reclaiming self-determination and identity for First Peoples after two centuries of denial of these important determinants of wellbeing by mainstream society. In this study, ‘First Peoples’ is the preferred term to refer to the Aboriginal Torres Strait Islander population, as it encompasses both the diversity of Aboriginal and Torres Strait Islander peoples in the region, while also reflecting the shared experiences that unite Indigenous peoples in Australia and internationally.

The *Creating Healthy Environments* project evolved from a history of collaboration between The University of Melbourne, Viney Morgan Aboriginal Medical Service, a primary health service focusing on clinical and social health; Rumbalara Football Netball Club, a sporting club that engages community in health promotion; and Rumbalara Aboriginal Co-operative, a multi-faceted organisation providing health, housing, emergency relief and Elder services [3]. The three ACCOs run a range of activities that promote healthy behaviours, physical activity, healthy lifestyle choices and nutrition awareness, and the University has collaborated with them on various teaching, research and evaluation projects. Rumbalara Football Netball Club stands out by focussing on promoting healthy lifestyles and preventative health programs rather than crisis intervention services [4]. All three ACCOs identified that their programs were not being evaluated and reported in ways that reflect how they address social determinants of First Peoples’ health and the holistic nature of their design [5]. This is not unique to this region or country [6–8]. Each of the ACCOs were locked into certain funding arrangements (which largely neglected prevention activities) that prevented them working together to achieve common goals. In 2010 the organisations formed the Goulburn-Murray Health Promotion Alliance. This alliance emerged from community leaders’ concerns regarding a lack of understandings about the impact their programs were having on the community, and how services could be better delivered. The project was the first of its kind in the region to challenge First Peoples’ organisations to examine what they were providing, and also the design and outcomes of their services. They were also interested in knowing if any of the services provided in the community organisations overlapped and if there were opportunities for collaborative program delivery. This was seen as a way of better utilising already under-resourced organisations in the region.

Thus the Creating Healthy Environments project was developed in part to address the limitations of monitoring and reporting health program activity [7]. We have previously used an ecological model as an indicator of health program complexity and design [5, 9] and found it to have value for this purpose, despite certain limitations [10]. In theory, health programs are more likely to be effective if they act to change the environment in which people live as well as working with individuals. Interventions following an ecological approach emphasise the relationship between people and the physical and social systems within which they live, including their social networks, organisations, communities, and the broader society. In this way, ecological models may be viewed as more closely aligned with First Peoples’ world views and holistic models of health, and therefore hold greater value for First Peoples and ACCOs. According to ecological theory, projects that intervene at many of these levels offer greater potential for promoting health over the short and long term than those with a single focus [11]. This approach is increasingly being acknowledged as a means of promoting health among First Peoples in Australia [12–14]. However, beyond interventions for preventing disease, ACCOs are important contributors to cultural identity and community development for First Peoples as noted above. We have previously identified important components of wellbeing that inform the cultural safety and service delivery practices of ACCOs in the region [2] but these aspects of wellbeing have not been systematically examined as part of program evaluation.

The initial aims of this project were to: 1) identify the aims and purpose of existing health promotion programs conducted within the Health Promotion Alliance; and 2) evaluate the extent to which these programs are consistent with an ecological model of health promotion, addressing both individual and environmental determinants of health.

This work was carried out with approval from The University of Melbourne’s Human Research Ethics Committee [0931708] and the Aboriginal Health and Medical Research Council and in line with ethical guidelines of the National Health and Medical Research Council of Australia [15].

## Methods

### Establishing the Health Promotion Alliance

Partnership, good communication, and trust were fundamental in working with the community organisations and especially if working in research [16]. The current work grew from a long history of collaboration with an emphasis on community direction, capacity development and exchange, a participatory research approach, and privileging First Peoples’ knowledge [2, 3, 17]. The project was funded by The Lowitja Institute and the NHMRC and included several community-based researchers as Chief Investigators. A Memorandum of Understanding guided the conduct of the research, which was overseen by a Steering Committee on which each of the organisations was represented. This manuscript was submitted for publication with approval from the Steering Committee.

The three community organisations provided an overview of the health activities and programs that were operating from their centre. The information provided an opportunity to map what each organisation was delivering to community members in health promotion [4]. Each organisation participated in the Alliance by nominating programs on which they felt comfortable collaborating and wanted to know more about. The detailed information collected prospectively at each partner organisation related to the following 12 programs: at Viney Morgan AMS, *Sister Girl* and *Ladies’ Art and Craft* women’s wellbeing programs; at RFNC, *Makin’ A Move* physical activity and nutrition program, *Fruit Share* and (with RAC) *3 Healthy Messages* programs for youth, *Unity Cup* reconciliation activities, and *RFNC Mural* community history project; at RAC, *Lulla’s Health Check* child health activity, *Divine Breast Day* women’s activities, *Koori Maternity Services* maternal and child health program, and *Elders’ Exercise Group* for senior community members. These programs do not represent the entire range of services delivered by the collaborating organisations, but are those that they decided to include as part of the Alliance’s body of work.

### Data collection and analysis

Local Indigenous workers were engaged and trained in gathering data, and to develop a set of tools that were culturally sensitive for the organisation to use in their programs beyond the life of the research project. The researchers worked with the organisations, their practitioners and program managers to discuss the process of collecting data and the tool used to gather the information on the health promotion programs prospectively, as described below.

**Fig. 1:**
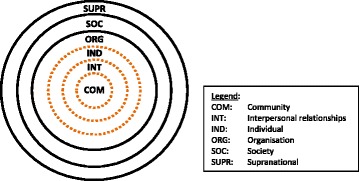
Working model of Aboriginal systems used to categorise the settings and targets of health promotion activities

Using a standardised form based on that used for the Kahnawake Schools Diabetes Prevention Program [18], with some modifications for local use, we collected information about 88 separate activities run as part of the health promotion programs listed above as they were being implemented, between 2011 and 2014. The form includes information about: activity name, sponsoring organisation, date, description; whether it fulfilled its objectives on the day; target group, numbers of participants and where they were recruited from; health focus; role of local culture in activity design; barriers and facilitators to implementation of the activity. We assessed the degree to which this group of programs integrated an ‘ecological’ approach considered best practice in health promotion, that is, how this program of health promotion addressed a range of individual and environmental determinants of health. For this purpose we then coded these activities by identifying a) where the participants were recruited from (the settings), b) who or what was meant to change as a result of the activity (the targets), and c) how this change was to be brought about (the strategies) [19]. Strategies are coded by linking a health promotion activity [HP] to its targets. For example, an activity that seeks to provide knowledge directly to participants is coded as HP → IND, while an activity that brings people together to strengthen their social connections is coded as HP → [IND-IND] → INT, and so on. Richard and colleagues [19] provide a method for categorising settings and targets within the levels of a nested hierarchy—individuals, interpersonal relationships, organisations, community, the broader society, and the supranational level. We have found that this systems model of the relationship between people and their environment, described in most detail by Miller [20], inaccurately describes First Peoples’ communities and we utilised an alternative model for the purpose of coding health promotion activities. It is beyond the scope of this paper to provide a detailed explanation of how this model was derived, but it is based on a series of in-depth discussions and workshops with Indigenous peoples and on other published work, including the ‘Living Communities’ model [21–23]. In our revised system, individuals, families, culture, identity and land are inextricably linked as a living community, not in a hierarchical manner but as interrelated and equal parts of an embodied whole. For coding, ‘culture’ and ‘identity’ are placed within the interpersonal (INT) category (‘culture’ being primarily the way in which people relate to each other, and ‘identity’ arising in large part from a person’s relationship to family, community and place) and Land within the community (COM) category. We have replaced Richard’s solid lines separating these levels with dotted lines to emphasise this non-hierarchical, fluid structure (Fig. 1). Organisations are placed at the interface between the First Peoples’ community and the broader Australian society (itself made up of many communities in addition to First Peoples). First Peoples’ community-controlled organisations (ORG) are expressions of self-determination, and many have evolved to become a bridge between community members and mainstream government, funding and professional institutions. Mainstream organisations (ORGm) emerge from the societal level and are an important point of contact between First Peoples and other communities and society. We recognise that First Peoples’ communities are among many regional, ethnic and social communities living within the Goulburn-Murray Rivers Region and making up Australian society, while holding a special place as the original inhabitants, Traditional Owners, and holders of cultural authority for this Country. First Peoples seek to be treated as equal, productive and leading communities within society, not as a ‘problem’ nor as ‘disadvantaged’. However, for the purpose of this analysis, First Peoples’ community is distinguished from other regional communities which are collectively (and very imprecisely) referred to as part of ‘mainstream’ society as there are unique social and political circumstances that affect the current relationship of First Peoples with Australian society and which impact negatively on health.

Unlike Richard and colleagues, and consistent with the community development philosophy of health care favoured by ACCOs [24], we have not assumed that the individual (IND) is the ultimate target for all health promotion activities, and this is reflected in the way strategies are coded. Finally, reflecting the overwhelming influence the large, majority non-Indigenous population [25] has on the cultural, social and economic wellbeing of First Peoples, the broader Australian society (SOC) is considered a legitimate target for change. This is necessary given the existence of ongoing discriminatory regional or national government policies, the high incidence of structural and personal racism [26] and the absence of a legally binding agreement between the government and the sovereign First Peoples. Coding for all activities, was performed by two authors (JD, KR) with additional input from staff of respective programs as required.

Descriptive statistics are presented showing frequencies of participant contacts by program, the health focus of the 88 health promotion activities implemented across the 12 programs, and of program settings, targets and strategies. Statistical analyses were performed using SPSS Version 22.

## Results

### Program areas and participant contacts

There was a wide range of activities addressing a number of environmental and social determinants of health, as well as physical activity. The programs assessed for this evaluation were, at Viney Morgan AMS:

*Sister Girl program*, which provided a safe and cultural space for community members to participate in activities that encouraged lifestyle changes in order to improve health and well-being, throughout the program and more widely. Over a period of 10 weeks the women would participate in activities in the gym, discuss healthy eating, sharing stories and, most importantly, support each other. The program thus promoted social and cultural connectedness between women, participants’ health knowledge and behaviour, and increased access to services (in this case, a gym) through organisational networking.

*Ladies’ Art and Craft* program sessions gave women the opportunity to come together and experience new ideas through their talents in jewellery making, sewing and other craft activities. Older women shared their knowledge with younger women, encouraging and supporting each other. The setting and the activities themselves were an important factor in bringing women out of their homes and into a safe place. It also provided women the space and time to talk about issues of health, family, budgeting, cooking—there were no boundaries attached. Hence social and cultural connectedness was a major aim of this program also, supported by the creation of a safe environment in which knowledge was shared.

At RFNC: *Makin’ A Move,* a fitness and weight loss program initiated by RFNC for community members. The 10-week program had two fully trained Fitness Instructors as co-ordinators (one from University of Melbourne, one from RFNC). Community and family members took part in walking laps around the oval and joining in group exercise classes at a local mainstream gym, and participated in discussions about ways to achieve better nutrition. The program also incorporated elements of the ‘My Moola’ financial literacy program. In addition to increasing participants’ knowledge of nutrition and supporting physical activity (including through networking between organisations), the program strengthened social and cultural connections through group activities.

The *Fruit Share* program provided fruit at RFNC on training nights so that Club members and players could choose healthy snacks before training. The activities particularly targeted junior players arriving at the ground after school so they had enough energy to train effectively. In this way the RFNC environment was modified to support healthy eating among club members.

The annual *Unity Cup* football and netball matches between RFNC and Congupna FNC aimed to promote social inclusion through reaffirming, celebrating and taking pride in First Peoples among all the communities of the region, showcasing the value of community to government at all levels, honouring all women and Elders and supporting young peoples’ role in community. Although an annual event, the *Unity Cup* leads to sustained change through the creation of new social norms of behaviour. The theme, ‘Honouring the Role of Women in our Communities’, was reflected in a very special spiritual performance, that allowed all women and children present on the day to walk side by side across the oval as local Elders carried burning gum leaves that represented the connection to country. As well as football and netball matches, activities held as part of the event included the sporting teams’ President’s lunch, with guest speakers from community and local and state governments. The activities of Unity Cup thereby sought to strengthen a very broad range of influences on First Peoples’ wellbeing—relationships with other community members, cross-cultural relationships with members of other local communities through promoting recognition of First Peoples’ culture within the football/netball league, and attitudes in the broader society through engagement with government.

The *RFNC Mural* was developed for display in the social rooms of the club in collaboration with the Yarrwul Nyuwandan Social Inclusion Project. The wall is a visually inspiring cultural statement of ‘Survival and Strength!’ It weaves together just a glimpse of the many representations that strongly connect us to our Dreaming. The wall honours First Peoples’ ways of knowing, seeing and doing, and it acknowledges and values our right to keep our spirituality on going since time began. This program promoted cultural maintenance and development within community through enhancing the environment at RFNC.

The *Three Healthy Messages* project, a collaboration with RAC, engaged several youth of the RFNC community to propose ways of promoting healthy living ideas to other youth. In a series of workshops they talked about different issues relevant to them as young people and also the effect of those on their family and community. The group identified three issues in health—binge drinking, smoking (tobacco use), and other drug use (particularly cannabis). The young participants brainstormed ideas and generated messages that were then communicated to other young people in the form of a short film clip as the end result of the whole program [27]. They also produced some promotional material, including posters and t-shirt designs. The participants were introduced to script writing and multimedia techniques to produce the film clip. The project developed the capacity and skills of the individual participants and the organisations involved, their social and cultural connections, and sought to influence community and society through posting the film clip on the world wide web.

At RAC: *Lulla’s Health Check Day*, a Children’s health check day conducted by RAC Health Promotion Unit. Parents were invited to attend with their 3 and 4 year old children. The checks were completed in 5 steps and included general measurements like height and weight, Parents’ Evaluation of Developmental Status [28], hearing checks, and eye testing. The health checks aimed to strengthen children, families and community.

The *Divine Breast Day* activity invited women from the community to join together to enjoy a day of pampering, yarning and education on breast health. The room was decorated in pink, purple and silver with lots of donated bras hanging everywhere. Women were encouraged to dress in pink and to bring an old bra with them. Prizes were given out for the best-dressed and the oldest bra and the judging was done by women. This broke the ice and women became comfortable to yarn and tell their stories and share history of their journey related to breast cancer. This was a very powerful and moving experience for all the women. Lunch was provided which gave the women more time to connect with each other and to ask further questions of the professionals present. Bookings were taken for breast screening on the day and followed up with reminder calls, transport and follow ups. The activities aimed to improve health for women through providing them with information both directly and through connecting with other women, and by increasing service accessibility through networking between organisations.

The *Koori Maternity Services* (KMS) team ran a cooking program called Winyarr (meaning “Aboriginal Women”) in which women came together and cooked healthy foods, shared ideas and connected over a meal. KMS also ran a belly-casting event for mothers and babies. This activity provided the mum-to-be with a special connection to the new baby. These activities thus aimed to strengthen families through working with mothers.

The *Elders’ Exercise Group* provided Elders, all of whom live in full time care, a fun and interesting way to interact with each other, help maintain their movement and strength, and provided a space for Elders to interact on a social and emotional level through conversation, stories and lots of laughter. The coordinator developed activities with the use of light hand weights, different size balls, stretching and balance. As well as promoting participants’ physical fitness, these activities were important for promoting social connections and for passing on cultural stories within the community.

Table 1 shows the number of activities analysed per program and the demographic characteristics of each program’s target groups. Across the programs making up the Health Promotion Alliance activities, all ages were represented, as were Elder and family groups. While several programs were specifically designed for women, and others for youth or infants, none were specifically for males (although a number did include male participants, and the Unity Cup football match is played by men).

**Table 1:** Programs and estimated participant contacts

Program name	sponsoring organisation	No. of activities	Participant contacts						
			Infants	children	youth	adults	Total	Elders	Families
			<5 years	5–12 years	13–24 years	25 years+			
Sister Girl	VMAMS	19	0	0	0	72	72	Y	Y
Ladies’ Art & Craft	VMAMS	8	1	0	4	34	39	Y	Y
Makin’ a Move	RFNC	31	0	0	22	97	119	N	N
My Moola	RFNC	3	0	0	1	8	9	Y	N
Fruit Share	RFNC	2	1	20	40	0	60	N	N
History Wall development	RFNC	2	2	1	0	19	22	Y	N
Unity Cup	RFNC	6					400 + ^a^	Y	Y
Lulla’s Health Check	RAC	1	35	0	0	0	35	N	Y
Divine Breast Screen	RAC	1	0	0	7	49	56	N	N
Koori Maternity Services	RAC	2	0	0	0	3	3	N	N
Elders’ Exercise Group	RAC	8	0	0	0	38	38	Y	N
3 Healthy Messages	RFNC/RAC	5	0	0	19	0	0	N	N

^a^not including mainstream community members

### Ecological analysis

Table 2 shows the various strategies used across the 12 programs, coded using the modified ecological coding procedure as described in Methods. A variety of strategies were used to address the program aims, most of which were based on strengthening relationships between people, and sometimes between organisations within First Peoples’ community and in the broader society. Using this method we found that strategies used in these health promotion programs could be categorised into five themes based on their ultimate target. These were:

**Table 2:** Frequencies of intervention strategies for 88 activities across 12 programs

Strategy	Frequency	Example
HP → [IND-IND] → INT → COM	9	Bringing Elders together in the *Elders Exercise Group* to exercise, share stories for maintaining culture in community
HP → IND → INT → ORG → COM	2	Weaving raffia for *RFNC Mural*, maintaining culture, developing RFNC environment, for community development
HP → IND → INT → COM	1	*Lulla’s Health Check Day* to assess children, build capacity of carers, leading to stronger Aboriginal community
HP → INT-ORG-COM	1	Aboriginal youth develop *Three Healthy Messages* film clip for community
HP → [IND-IND] → INT	53	Bringing women together to strengthen social connections in *Sista Girl*
HP → IND → INT	10	Learning to shop on a budget for the family in *Makin a Move*
HP → INT	4	Sharing knowledge in group via social networks at the *Devine Breast Day.*
HP → [COM-COM] → INT	1	Women from 2 communities meet to strengthen social connections in *Ladies Art and Craft*
HP → [ORG-ORGm] → INT	1	Presentation of the *Unity Cup*, bringing together RFNC and CFNC to strengthen social relationships
HP → [ORG-ORGm] → ORGm → INT	1	Bringing together RFNC and CFNC for the *Unity Cup*, to promote cultural competence in the Murray Football League
HP → IND	71	Increasing knowledge of health, nutrition, financial literacy in *Makin a Move*
HP → [ORG-ORGm] → INT → IND*	21	Koori orgs and mainstream gyms work together to create a culturally safe environment for physical activity in *Sista Girl*
HP → INT → IND	4	Strengthening relationships between younger and older women to promote breast health at the *Devine Breast Day*
HP → [INT-INT] → IND	1	Unity cup football match, promoting wellbeing among men
HP → ORG	8	Creating a culturally safe space for community women in *Sista Girl*
HP → [IND-IND] → INT → ORG	4	Training young people to produce *Three Healthy Messages* film clip
HP → IND → INT → ORG	2	*Fruit Share* provided for RFNC junior players to strengthen team and club
HP → [IND-IND] → ORG	1	Participants provide input on program design in *Three Healthy Messages*
HP → COM, SOC	1	Aboriginal-produced *Three Healthy Messages* film clip uploaded to the internet
HP → INT → COM, SOC	1	Guard of honour for women at *Unity Cup*, strengthening Aboriginal and mainstream culture
HP → [INT-INT] → COM, SOC	1	Senior football teams play for *Unity Cup* to strengthen community and mainstream society
HP → [IND-IND] → INT → SOC	1	Aboriginal and mainstream community members come together at *Unity Cup* to promote recognition of First Peoples

Legend: *HP* Health Promotion Program, *IND* Individual- eg. individual knowledge, behaviour, *INT* Interpersonal- eg. relationships between people, social connection, culture, identity, *ORG* Organisation. Brackets [ORG-ORG] represents the creation of a link between organisations, *ORGm* Mainstream Organisation, *COM* Community- meaning both people united by geography or culture, as well as associated physical environments, *SOC* The broader, mainstream society, *Square brackets* Indicate bringing together or forming links between

Cultural maintenance and community development,^1^ in which the First Peoples’ community [COM] is the ultimate intended beneficiary of the activities. For example: activities at RFNC such as the History Wall were specifically about strengthening collective cultural identity via working with groups of participants [IND, INT] to develop the organisational environment [ORG], and RAC Elders’ group [IND-IND] exercise program included elements of sharing stories and connections [INT] for cultural maintenance;

Social and cultural connectedness, which involved (re)connecting to strengthen culture, spirit, family and identity as a priority outcome. These fall within the interpersonal [INT] domain in our revised scheme (Fig. 1, Table 2) and commonly occurred through implementing group exercise or art activities [IND-IND] for example, or through targeting families by building capacity of mothers [IND]. Activities targeting First Peoples’ relationship with organisations other than ACCOs and staff members were also present by increasing cultural safety at gyms and within the sporting competitions through organisational networking [ORG-ORGm];

Individual knowledge and behaviour such as providing information about diet and financial literacy at the individual [IND] level. This rarely if ever occurred in isolation from strategies that also aimed to strengthen social and cultural connections [INT];

Developing organisations and programs [ORG] to make them culturally safe, healthy and accessible for community members. For example, developing programs such as *3 Healthy Messages* and *Makin’ A Move* involved bringing participants together [IND-IND] to both strengthen relationships [INT] and to optimise program design;

Building cross-cultural relationships between the wider society [SOC] and First Peoples. Influencing societal attitudes to First Peoples is considered an important way to reduce health inequities, and the *Unity Cup* was one initiative that sought to achieve this through engagement with diverse community members, organisations and governments.

Thus, in terms of the ultimate intended target of program strategies, for 49 % of observed strategies, individual program participants were targeted, either directly by exercise and educational strategies or through personal networks and organisational partnerships. Strategies bringing individuals, communities or organisations together for strengthening relationships were also frequent (35 % of all activities)—most activities were group-based and creating and strengthening relationships between participants was an aim of these types of programs, while some activities aimed to give participants knowledge about healthy eating and recipes to take back to family. The majority of strategies included some form of social connectedness as part of their design. A substantial minority (7 %) of strategies were ultimately and explicitly about strengthening community into the future. A similar proportion sought to promote cultural safety within organisations (including organisations other than ACCOs) and several were aimed at changing societal attitudes.

Overall, four different types of proximal (initial) targets were identified, the majority of them (78 %) at the individual level. Sixteen percent of strategies directly targeted organisations or organisational relationships. For example, RFNC was identified as an organisational target through its partnership with other institutions (e.g. University of Melbourne) to develop and run programs like ‘Makin’ A Move’, and through providing a healthy eating environment through the ‘Fruit Share’ program. One activity as part of *Ladies’ Art and Craft* program aimed to strengthen the connection between two communities.

The programs assessed in this review recruited participating people and organisations from four types of settings: from the community (e.g. Cummeragunja residents participating in *Ladies’ Art and Craft*); from within the participating organisations (e.g. RAC clients in *Koori Maternity Services*); from within organisations (e.g. from a regional football league as part of *Unity Cup*); and from the broader society (e.g. by engaging local and state government organisations). Just under half of the programs recruited from a single setting, and this was most often the organisation (Table 3). Fifty percent of programs recruited from two or three settings. One program, *Unity Cup* at RFNC, recruited participants from four different settings, including multiple ACCOs and other organisations and the broader society. RFNC engages with mainstream organisations and society routinely through its involvement in sport and other activities and hence often recruits program participants from a range of settings. VMAMS, which is relatively isolated geographically, also engaged frequently with other settings including access to gyms. RAC most often recruited from within its own organisation, as it is located in a population centre and services a large client base.

**Table 3:** Frequency of programs (*n =* 12) according to number and types of intervention setting

No. of settings	Frequency	Types
One	5	community (1); organisation (4)
Two	4	organisation, mainstream organisation (1); community, organisation (4)
Three	2	community, organisation, society
Four	1	community, organisation, mainstream organisation, society

Collectively, this represents a very good design for a program of health promotion, with multiple settings, a range of individual and environmental target types, and numerous and innovative strategies.

### The role of culture in program design

**Fig. 2:**
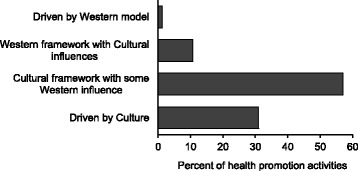
Influence of culture in the design of health promotion activities

The design of the great majority of activities implemented as part of the Health Promotion Alliance had minimal Western influence and were designed within a local Aboriginal cultural framework (Fig. 2). This reflects the community-controlled nature of most of the programs and the fact that local community workers designed and ran them from within the ACCO sector to meet the needs of their members.

### Wellbeing focus of program activities

**Fig. 3:**
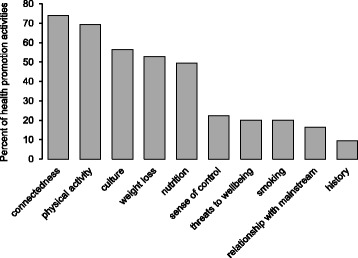
Wellbeing focus of program activities

Program activities were characterised according to whether they focussed on a range of commonly funded health promotion priorities, and previously identified aspects of wellbeing for Aboriginal people in the region [2]. The most common focus of the health promotion activities was social connectedness, which was recorded as a focus for three quarters of the activities across the 12 programs. Physical activity was represented in two thirds of the activities, and nutrition, weight loss and culture were each a focus of about half of the activities. Increasing participants’ sense of control, cigarettes and other threats to wellbeing, acknowledging and celebrating history, and strengthening relationships with mainstream society were a focus of some activities (Fig. 3). Elements of social connectedness were almost always incorporated in the program design of those focusing on nutrition, physical activity and weight loss (data not shown).

## Discussion

This prospective study of First Peoples’ health promotion showed that these Aboriginal organisations are providing culturally relevant and innovative health activities that address community needs for First Peoples of the Goulburn-Murray Rivers Region. The aims and purpose of these health promotion activities were much broader than for conventional programs. The design was consistent with a First Peoples’ ecological model of health promotion.

### Aims and purpose of first peoples’ health promotion

At its simplest level, health education is about giving people knowledge about their health and how to improve it. This is important but, on its own, is the least likely strategy for successful long-term change in health for individuals, families and communities. Furthermore, it does not necessarily achieve the aim of health promotion which is to enable individuals and communities to increase control over their health and its determinants [29].

In this study we have documented a wide range of activity addressing a number of individual and social environmental determinants of health. The health promotion programs we have studied included a mix of different types of targets: individual knowledge and behaviours; community development; social connectedness; organisational development; and First Peoples’ relationship with the broader Australian society. Participants were recruited from a range of settings including ACCOs and other organisations and society. Health promotion activities involved all age groups from infants to adults and included programs targeting Elders and families. Thus First Peoples’ health promotion is complex in design and inclusive, consistent with an ecological approach. The ecological analysis showed that the programs implemented by the Health Promotion Alliance worked not only with individuals and their lifestyle choices but with all components of the Living Communities model [23]—people, culture, identity and place—as well as First People’s and other organisations and broader Australian society. Furthermore, it included strategies that concentrated on community development as the goal of program activities. Others sought to improve the collective status of First Peoples in a frequently hostile society. Social connectedness was highly prominent in program design, both as an outcome in itself and as a long-term strategy for achieving change in other areas, and with resulting flow-on effects into the community. This approach by the participating ACCOs is consistent with the comprehensive primary health care model as developed by First Peoples in the 1970s [1, 24].

The Health Promotion Alliance was made up of a medical service focusing on clinical and social environmental health; a co-operative providing a range of services in health, housing, emergency relief and Elders services; a sports club which conducts healthy lifestyle programs; and a university department providing evaluation expertise. For programs included in the Health Promotion Alliance, the community organisations differed in their recruitment focus with their programs recruiting from settings within the organisation or community, and targeting different age and social groups of the community such as mothers and children, youth and Elders. The various programs were thus complementary to each other (although specific aspects of men’s wellbeing were scarce Nevertheless, joint activities across organisations were uncommon, with the *Three Health Messages* being a notable exception. This activity was implemented towards the end of the evaluation period, and is a hopeful indication of stronger possible linkages between agencies in the future.

### The focus of health promotion activities

The Health Promotion Alliance included program activities in a broad range of social, cultural, clinical and environmental areas. Nutrition, physical activity and weight loss were prominent, but so too were art, culture and strengthening relationships between people and communities. This breadth reflects the goals, structure and funding of the different partner organisations. All of the programs included in this study incorporated primary prevention activities, as opposed to working with people who are already unwell, with an emphasis on increasing knowledge, maintaining social connections, culture, and physical activity. This emphasis is consistent with strengthening the resilience of the community and its members as a way of preventing illness and promoting wellbeing [1]. However it is not necessarily what the organisations are specifically funded for in all cases, which often focusses on a small number of specific outcomes such as weight loss or smoking cessation. Further investigation, systematically comparing funders’ aims and community aims of health promotion, is required to comprehensively describe and recognise gaps in understanding.

It is significant that a large proportion of activities focussed on determinants of wellbeing that have been specifically targeted for elimination by the colonising society since invasion—social connectedness, culture and sense of control. Deliberate destruction of family and tribal connections, particularly between generations, the replacement of First Peoples’ culture with European values and norms, and control of most aspects of First Peoples’ lives were overt policies of successive governments [30] and appear to remain part of the collective psyche of Australian society. Thus First Peoples’ health promotion is in part about reclaiming and strengthening these major drivers of health and wellbeing [31].

### The importance of First People’s culture in health promotion program design

There is need to understand the connections and the culture of these organisations and, specifically, who controls what is delivered to community. As government funding supplies the material resources they also have a vested interest in how money is being spent on First Peoples’ well-being, and in return require constant reports back to the relevant Department. All the programs included in this study were designed and run by local community members within ACCOs, with a couple of exceptions where activities were implemented by other organisations. Thus an ecological approach intrinsically emerged during the program, and social connections and social determinants were incorporated and demonstrated in activity design. Therefore, though these issues may or may not have been explicitly recognised in the initial program design, they became consistent with the theoretical best practice in health promotion design over the course of the program.

Each program’s design was informed by First People’s “ideas, customs, and social behaviour” (that is, “culture”) [32]. Routine reporting does not capture these cultural aspects of program design and hence programs are not often funded or evaluated against cultural design aspects or outcomes. Overlooking these important design elements prevents Australian society from benefiting from the leadership in health promotion as demonstrated by First Peoples, as we have described here. Kenny has discussed the challenges for First Peoples providing leadership in many areas when they are required to walk between their own culture and the dominant culture [33].

Consistent with our observations, First Peoples’ health promotion more generally addresses a broad range of individual, social and environmental influences on health. Thus a recent issue of the *Australian Journal of Primary Health*, focussing on health promotion in Aboriginal and Torres Strait Islander communities, included reports on: smoking cessation through social marketing [34]; trachoma elimination using Australian Football League clinics to promote health and hygiene [35]; using participatory action research to prevent suicide in Aboriginal and Torres Strait Islanders communities [36]; building social connectedness through an Aboriginal community football club [37]; healthy food choices and nutrition promotion in a remote community [38]; and addressing employment discrimination [39].

### Evaluation of first peoples’ health promotion programs

We have used an ecological framework in this and other projects to try and capture more accurately the complex nature of First Peoples’ health promotion. The Living Communities model [23] has influenced our evaluation methods by bringing a First Peoples’ perspective to existing Eurocentric models [19]. To a certain extent, this model has allowed us to systematically describe the breadth and complexity of First Peoples’ health promotion in this region. Our modified coding scheme has also allowed a better description of the community development and sociocultural aspects of the organisations’ activities than does the original coding scheme which was designed for mainstream disease prevention programs [19]. Even so, the method gives only a simplified description of program design and purpose and it may require further development [10].

This evaluation method has highlighted the breadth of health promotion program aims in this setting. This is important because funding of activities and acknowledgement of their many interrelated outcomes need to consider this breadth and complexity. Failing to do this perpetually creates a loss of control for the organisations to allocate funding as appropriate for the community. The skill set to implement this range of activities also needs to be acknowledged and supported. Organisations have found it difficult to provide Aboriginal staff with opportunities to increase their skill base through training programs, or even encourage workers to take on large amounts of training that will offer diplomas or further education. Finally, there are issues for Aboriginal employees in mainstream workplaces. These relate to expectations on them and the limitations of what they can do in an environment that does not recognise the importance of the living communities model in the design of health promotion for First Peoples.

### Limitations of this study

The health promotion programs included in this study are a non-random sample of the bigger program of work currently established by the partner ACCOs. In this sense they may not be wholly representative of health promotion in the region. However, for the purpose of describing the breadth and aims of First Peoples’ health promotion, they provide an important insight into a previously under-researched topic. We have not monitored trends in ‘outcomes’ as part of this project as it was not part of the project’s aims, which instead focused on program design, purpose and implementation. We view this process as an important step towards also developing better measures of the effectiveness of First Peoples’ health promotion programs, using measures that appropriately reflect First Peoples’ priorities, values and models of health.

Several of the terms used to describe activity focus—specifically, ‘culture’ and ‘sense of control’—were not precisely defined and in interviewing practitioners some degree of interpretation on their part was required. Many activities can fall within the definition of ‘culture’ and we chose to let program practitioners define whether the activities they ran had the specific aim of strengthening local culture rather than impose our own definitions. ‘Sense of control’ has many individual and collective aspects, and all require further investigation into their salience for First Peoples [2] but, again, we deferred to practitioners’ knowledge of program activities as to whether increasing participants’ control or self-efficacy was part of the aim.

### Future work

The development of tools for practitioners to monitor, evaluate and report their own programs is required and was a key aim of the project. At this stage, a modified version of the activity implementation form currently being used by research staff seems to be the most feasible tool for further development. The event log method [40] would also be extremely valuable as a tool for both evaluation and reflexive practice if practitioners were prepared to use it. This would require both the interest of the practitioners, and the involvement of program managers to ensure that the event logs were built into organisational processes rather than perceived as an additional burden. As the Alliance continues to work together, the integration of event logs into practice will continue to be explored.

To create partnerships locally with our partner organisations there is a need to build Health Promotion Alliance programs that nurture and strengthen each other. This requires conversation, connecting, planning, trust and targets to move forward together. Work in other areas has demonstrated that practitioners and organisations need to feel some ownership over a project in order to engage closely with it [41]. Clearer and more frequent communication from the research team is an important step towards this. Collaborative projects like *3 Healthy Messages* that bring us together regularly to plan and implement health promotion activities are emerging and are a focus for stronger partnership.

## Conclusions

First Peoples’ health promotion in the Goulburn-Murray Rivers region encompasses a broad range of social, cultural, lifestyle and community development activities including reclaiming and strengthening cultural identity and social connectedness as a response to colonisation. Social connectedness was a major component of a majority of activities that were implemented as part of the Health Promotion Alliance, including important parts of activities addressing nutrition, physical activity and weight loss.

At present, there are barriers, such as limited time or capacity, that lead to First Peoples perspectives being excluded from health promotion implementation and evaluation processes, resulting in inaccurate or misleading conclusions based on available data. The present study seeks to move towards understanding how First Peoples’ health promotion is implemented, in line with community priorities and processes, and to thereby improve the ability of mainstream institutions to grasp and respond to these priorities. In order to achieve sustainable program implementation and monitoring of outcomes, funding processes need to move beyond short-term cycles that increase competition rather than collaboration. Evaluation and reporting of First Peoples’ health promotion should be aligned with the important social and cultural aims and practices that are integral to health promotion programs for First Peoples.

## Abbreviations

ACCO: Aboriginal Community Controlled Organisation
CRC: Co-operative Research Centre
NHMRC: National Health and Medical Research Council
RAC: Rumbalara Aboriginal Co-operative
VMAMS: Viney Morgan Aboriginal Medical Service

## Footnotes

1 ‘Community development’ includes social and physical resources, capacity and the recognition by society of First Peoples.
